# Implementation of an Enhanced Crayfish Optimization Algorithm

**DOI:** 10.3390/biomimetics9060341

**Published:** 2024-06-04

**Authors:** Yi Zhang, Pengtao Liu, Yanhong Li

**Affiliations:** 1 College of Electrical and Computer Science, Jilin Jianzhu University, Changchun 130000, China; lpt1203@hotmail.com; 2 Jilin Provincial Department of Human Resources and Social Security, Changchun 130000, China

**Keywords:** crayfish optimization algorithm, Halton sequence, quasi opposition-based learning, fish device aggregation effect, IEEE CEC2019

## Abstract

This paper presents an enhanced crayfish optimization algorithm (ECOA). The ECOA includes four improvement strategies. Firstly, the Halton sequence was used to improve the population initialization of the crayfish optimization algorithm. Furthermore, the quasi opposition-based learning strategy is introduced to generate the opposite solution of the population, increasing the algorithm’s searching ability. Thirdly, the elite factor guides the predation stage to avoid blindness in this stage. Finally, the fish aggregation device effect is introduced to increase the ability of the algorithm to jump out of the local optimal. This paper performed tests on the widely used IEEE CEC2019 test function set to verify the validity of the proposed ECOA method. The experimental results show that the proposed ECOA has a faster convergence speed, greater performance stability, and a stronger ability to jump out of local optimal compared with other popular algorithms. Finally, the ECOA was applied to two real-world engineering optimization problems, verifying its ability to solve practical optimization problems and its superiority compared to other algorithms.

## 1. Introduction

Engineering problems have become increasingly complex [1] with the rapid development of engineering technology. As a result, more and more issues need to be optimized, making the importance of optimization increasingly prominent [2]. Traditional optimization algorithms, such as linear programming [3], gradient descent [4], and the simplex method [5], are often used to solve these problems in the past. Although they performed well in solving simple problems, their limitations became more apparent as engineering challenges and data processing requirements increased. Traditional algorithms are difficult to solve high-dimensional, nonlinear, or discrete optimization problems. Therefore, the swarm intelligent optimization algorithm [6] was developed, simulating the behavior of biological groups in nature to seek the optimal solution through cooperation and information exchange between individuals [7]. The swarm intelligence algorithms have more vital global optimization ability, robustness, and adaptability and can deal with various complex optimization tasks compared to traditional algorithms. These problems are all complex, and a swarm intelligent optimization algorithm can help find the optimal or approximate optimal solution [8], improving the solving efficiency and solution quality.

Many swarming optimization algorithms have been proposed recently due to their simplicity and strong optimization capabilities [9]. These algorithms include ant colony algorithm [10] (ACO) inspired by the foraging behavior of ants in nature, particle swarm optimization algorithm [11] (PSO) that simulates bird foraging behavior, and whale optimization algorithm [12] (WOA) that simulates the unique search methods and trapping mechanisms of humpback whales, among others. Although these standard swarming algorithms have been successful in many problems, they may still face some challenges in some cases [13]. Scholars have proposed several improved versions to address these challenges and improve the performance of swarm intelligence algorithms. For instance, Hadi Moazen et al. [14] identified the shortcomings of the PSO and developed an improved PSO-ELPM algorithm with elite learning, enhanced parameter updating, and exponential mutation operators. This algorithm balances the exploration and development capabilities of the PSO and produces higher accuracy at an acceptable time complexity, as demonstrated by the CEC 2017 test set. Similarly, Ya Shen et al. [15] proposed the MEWOA algorithm, which divides individual whales into three subpopulations and adopts different renewal methods and evolutionary strategies. This algorithm has been applied to global optimization and engineering design problems, and its effectiveness and competitiveness have been verified. Furthermore, Fang Zhu et al. [16] found that the Dung Beetle optimization algorithm (DBO) was prone to falling into local optima in the late optimization period. They proposed the Dung Beetle search algorithm (QHDBO) based on quantum computing and a multi-strategy mixture to address this issue. The strategy’s effectiveness was verified through experiments, which significantly improved the convergence speed and optimization accuracy of the DBO. These proposed and improved optimization algorithms significantly promote the development of swarm intelligence algorithms and make their performance better, allowing them to be applied to optimization problems in various fields, such as workshop optimization scheduling [17], microgrid optimization scheduling [18], vehicle path planning [19], engineering design [20], and wireless sensor layout [21].

The crayfish optimization algorithm [22] (COA) was proposed in September 2023 as a new swarm intelligence optimization algorithm inspired by the summer, competition, and predatory behavior of crayfish. There is intense competition in global optimization and engineering optimization. However, experiments have found that the convergence rate could be slower in some problems and has fallen into local optimal problems. This paper presents four strategies to improve it and comprehensively improve the optimization performance of the COA. The main contributions of this paper are as follows:

(1) An enhanced crayfish optimization algorithm (ECOA) is proposed by mixing four improvement strategies. Halton sequence was introduced to improve the population initialization process of COA, which made the initial population distribution of crayfish more uniform and increased the initial population’s diversity and the early COA’s convergence rate. Before crayfish began to summer resort, compete, and predate, QOBL was applied to the COA population, which was conducive to increasing the search range of the population and improving the quality of candidate solutions to accelerate the convergence rate. There is a certain blindness in this process, and elite factors are introduced to guide the crayfish because crayfish can directly ingest food when the size of the food is appropriate. This paper introduces the fish device aggregation effect (FADs) in the marine predator algorithm (MPA) into the predation phase of crayfish to enhance the ability of COA to jump out of local optimality.
(2) The proposed ECOA solves the widely used IEEE CEC2019 test function set and compares it with four standard swarm intelligence algorithms, four improved swarm intelligence algorithms, and crayfish optimization algorithms, respectively. Five experiments were carried out: numerical experiment, iterative curve analysis, box plot analysis, the Wilcoxon rank sum test, and ablation experiments. The experimental results show that the proposed ECOA is competitive and compared to similar algorithms, it has faster convergence speed, higher convergence accuracy, and stronger ability to jump out of local optima.
(3) Using the ECOA for practical optimization problems in the three-bar truss design and pressure vessels design, and comparing it with other algorithms. The ECOA shows higher convergence accuracy, faster convergence speed, and higher stability compared to other algorithms.

The rest of this paper is arranged as follows: Part 2 introduces the standard crayfish optimization algorithm (COA), Part 3 details the proposed enhanced crayfish optimization algorithm (ECOA), Part 4 tests the effectiveness of the ECOA and its superiority over other optimization algorithms through five experiments, Part 5 applies the ECOA to practical engineering optimization problems and Part 6 is the conclusion and future work.

## 2. The Crayfish Optimization Algorithm (COA)

The crayfish optimization algorithm (COA) is a novel swarm intelligence optimization algorithm inspired by crayfish’s summer heat, competition, and predation behavior. Crayfish are arthropods of the shrimp family that live in various freshwater areas. Research has shown that crayfish behave differently in different ambient temperatures. In the mathematical modeling of COA, the heat escape, competition, and predation behavior are defined as three distinct stages, and the optimization algorithm is controlled to enter different stages by defining different temperature intervals. Among them, the summer stage is the exploration stage of COA, the competition stage, and the foraging stage is the development stage of COA. The steps of the COA are described in detail below.

### 2.1. Population Initialization

The COA is a population-based algorithm that starts with population initialization to provide a suitable starting point for the subsequent optimization process. In the modeling of COA, the location of each crayfish represents a candidate solution to a problem, which has D dimension, and the population location of N crayfish constitutes a group of candidate solutions *X*, whose expression is shown as Equation (1).

(1) $$X=\left[\begin{array}{c} X_{1}\\ \vdots \\ \begin{array}{c} X_{i}\\ \begin{array}{c} \vdots \\ X_{N} \end{array} \end{array} \end{array}\right]={\left[\begin{array}{ccc} X_{1,1} & \cdots & \begin{array}{cc} X_{1,j} & \begin{array}{cc} \ldots & X_{1,D} \end{array} \end{array}\\ \vdots & \ddots & \begin{array}{cc} \vdots & \begin{array}{cc} \ddots & \vdots \end{array} \end{array}\\ \begin{array}{c} X_{i,1}\\ \vdots \\ X_{N,1} \end{array} & \begin{array}{c} \ldots \\ \ddots \\ \cdots \end{array} & \begin{array}{cc} \begin{array}{c} X_{i,j}\\ \vdots \\ X_{N,j} \end{array} & \begin{array}{c} \begin{array}{cc} \ldots & X_{i,D} \end{array}\\ \begin{array}{cc} \ddots & \vdots \end{array}\\ \begin{array}{cc} \cdots & X_{N,D} \end{array} \end{array} \end{array} \end{array}\right]}_{N\times D}$$

where X is the location of the initial crayfish population, N is the number of crayfish population, D is the dimension of the problem, and $X_{i,j}$ is the initial location of the i crayfish in the j dimension, which is generated in the search space of the problem randomly, and the specific expression of $X_{i,j}$ is shown in Equation (2).

(2) $$X_{i,j}={lb}_{j}+\left({ub}_{j}-{lb}_{j}\right)\cdot r,i=1,2,\cdots ,N;j=1,2,\cdots ,D$$

where ${lb}_{j}$ is the lower bound of the *j*-dimension of the problem variable in the search space, ${ub}_{j}$ is the upper bound, and r is the uniformly distributed random number belonging to [0, 1].

### 2.2. Define Temperature and Crawfish Food Intake

At different ambient temperatures, crayfish will enter different stages. The crayfish will enter the summer stage when the temperature is above 30 °C. Crayfish have assertive predation behavior between 15 °C and 30 °C, with 25 °C being the optimal temperature. Their food intake is also affected by temperature and is approximately normal as temperature changes. In COA, the temperature Temp is defined as Equation (3).

(3) Temp=20+r·15

where Temp is the ambient temperature. The mathematical expression of food intake P of crayfish is shown in Equation (4).

(4) $$P=C_{1}\cdot \left(\frac{1}{\sqrt{2\pi }\cdot \sigma }\cdot \exp\left(-\frac{{\left(Temp-\mu \right)}^{2}}{2\sigma^{2}}\right)\right)$$

where μ is the optimal temperature and $C_{1}$ and σ are used to control the food intake of crayfish at different ambient temperatures.

### 2.3. Summer Phase

When the temperature Temp is higher than 30 °C, crayfish will choose $X_{shade}$ cave for heat escape, which is the heat escape stage of the COA. The mathematical definition of $X_{shade}$ cave is shown in Equation (5).

(5) $$X_{shade}=0.5\cdot \left(X_{G}+X_{L}\right)$$

where $X_{G}$ is the optimal position obtained by the algorithm iteration so far, and $X_{L}$ is the optimal position of the current crayfish population.

There may be competition for crawfish to get into the heat. Multiple crayfish will compete for the same burrow to escape the heat if there are many crayfish and few burrows. This will not be the case if there are more caves. A random number between 0 and 1, rand is used to determine whether a race has occurred in the COA. When the random number rand<0.5, no other crayfish compete for the cave, and crayfish can directly enter the cave to escape the heat. The mathematical expression of this process is shown in Equation (6).

(6) $$X_{i,j}^{t+1}=X_{i,j}^{t}+C_{2}\cdot r\cdot \left(X_{shade}-X_{i,j}^{t}\right)$$

where t is the current number of iterations, $X_{i,j}^{t}$ is the current position of the *i* crayfish in the j_th_ dimension, t+1 represents the number of iterations of the next generation, r is a random number [0, 1], and the value of $C_{2}$ decreases with the increase in iterations, as expressed in Equation (7).

(7) $$C_{2}=2-\left(\frac{t}{T}\right),t=1,2,\cdots ,T$$

where T is the maximum number of iterations of the algorithm.

### 2.4. Competition Phase

Multiple crayfish will compete for a cave and enter the competition stage when the temperature Temp is higher than 30 °C and the random number rand≥0.5. At this stage, the position of the crayfish is updated, as shown in Equation (8).

(8) $$X_{i,j}^{t+1}=X_{i,j}^{t}-X_{z,j}^{t}+X_{shade}$$

where z is a random crayfish in the population, and its expression is shown in Equation (9).

(9) $$z=round\left(r\cdot (N-1)\right)+1$$

where r is the random number belonging to [0, 1], and $round\left(\cdot \right)$ is the integer function.

### 2.5. Predation Stage

The crayfish will hunt and eat food when the temperature Temp≤30 °C. The Crawfish move towards their food and eat it. The food location $X_{food}$ is defined in Equation (10).

(10) $$X_{food}=X_{G}$$

The crayfish will judge the size of the food to adopt different ways before ingesting food. The size Q of food is defined in Equation (11). The crayfish will tear the food with their claws first if the food is too large, and alternate eating with their second and third walking feet.

(11) $$Q=C_{3}\cdot r\cdot \left(\frac{{Fitness}_{i}}{{Fitness}_{food}}\right)$$

where $C_{3}$ is the food factor, representing the maximum value of food, and the value is constant 3; ${Fitness}_{i}$ is the fitness value of the *i* crayfish, that is the objective function value; ${Fitness}_{food}$ represents the fitness value of the food location $X_{food}$. The Crayfish judge the size of their food by the size $C_{3}$ of their maximum food. When the size of the food $Q>\left(C_{3}+1\right)/2$, the food is too large, and the tiny dragon will use chelates (shrimp claws, the first pair of feet) to tear the food; the mathematical expression is as Equation (12).

(12) $$X_{food}=\exp\left(-\frac{1}{Q}\right)\cdot X_{food}$$

After that, the crayfish will alternate feeding with the second and third feet, a process simulated in the COA using sine and cosine functions, as shown in Equation (13).

(13) $$X_{i,j}^{t+1}=X_{i,j}^{t}+X_{food}\cdot P\cdot \left(\cos\left(2\cdot \pi \cdot r\right)-\sin\left(2\cdot \pi \cdot r\right)\right)$$

where P is the food intake and r is the random number belonging to [0, 1].

The food size is appropriate and crayfish can be directly ingested when $Q\leq \left(C_{3}+1\right)/2$, and the position update expression is shown in Equation (14).

(14) $$X_{i,j}^{t+1}=P\cdot \left(X_{i,j}^{t}-X_{food}\right)+P\cdot r\cdot X_{i,j}^{t}$$

where r is a random number belonging to [0, 1].

## 3. The Enhanced Crayfish Optimization Algorithm (ECOA)

This section describes the proposed enhanced crayfish optimization algorithm (ECOA). Because the crawfish optimization algorithm (COA) has defects in slow convergence speed and easy falling into local areas, this paper adopts four strategies to improve it and comprehensively enhance the optimization performance of the crawfish optimization algorithm. Firstly, the Halton sequence is used to improve the population initialization so that the initial population is more evenly distributed in the search space. Secondly, quasi opposition-based learning strategy is introduced to generate the quasi-oppositional solution of the population, and the next-generation population is selected by greedy strategy, which increases the search space and enhances the diversity of the crayfish population. Thirdly, the foraging stage is improved, and the elite guiding factor is introduced to enhance the optimization rate of this stage. Finally, after the foraging stage, the vortex effect of the marine predator algorithm is introduced to strengthen the ability of the algorithm to jump out of the local optimal. Specific enhancement strategies are as follows.

### 3.1. Halton Sequence Population Initialization

Generally, population initialization in swarm intelligent optimization algorithms creates initial solutions that can converge to better solutions through continuous iteration [23]. This process forms the basis of algorithm iteration, and the population initialization quality directly affects the algorithm’s iteration speed and global optimization ability. In a standard COA, crayfish populations are generated randomly. Although this initialization method is simple and easy to implement, it may lead to uneven distribution of small and medium-sized lobsters in the population, and the population cannot cover the entire search space well, resulting in slow convergence of the algorithm and even falling into local optimal prematurely.

In contrast, the Halton sequence [24] makes the generated points evenly distributed throughout the search space as a low difference [25] numerical sequence. It differs from random sequences that create different points each time because of its deterministic nature. This paper uses the Halton sequence to replace the original random initialization strategy. This alternative can make the initial population cover the whole search space more evenly, improve the diversity of the population, and speed up the algorithm’s convergence. The expression for the initial location of each crayfish produced by population initialization based on the Halton sequence is shown in Equation (15).

(15) $$X_{i,j}={lb}_{j}+\left({ub}_{j}-{lb}_{j}\right)\cdot Haltonset,i=1,2,\cdots ,N;j=1,2,\cdots ,D$$

where Haltonset is a value based on the Halton sequence.

### 3.2. Quasi Opposition-Based Learning

Opposition-based learning [26] (OBL) was first proposed by Tizhoosh in 2005 and has been widely used to improve swarm intelligence optimization algorithms in subsequent studies. The concept is to generate the opposite solution of the current population, and by comparing the current solution and the opposite solution, retain the better candidate solution of the two as the next-generation population. OBL proposed that the opposition values of the current candidate solutions may be closer to the optimal solution, and the process is conducive to increasing the search range of the population and improving the quality of the candidate solutions. The position expression of the generated opposition solutions is shown in Equation (16).

(16) $$X_{i}^{OBL}=\left(lb+ub\right)-X_{i}$$

where *lb* is the lower bound of the problem variable in the search space, and ub is the upper bound.

**Figure 1:**
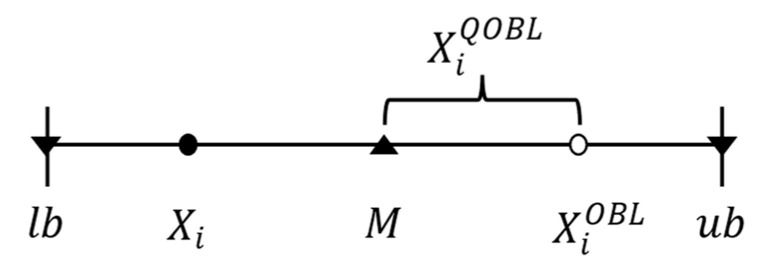
Schematic diagram of individual location of QOBL population.

There are also limitations. Inverse learning can improve the algorithm’s convergence speed in the early iteration stage, although some improvements show good performance. The inverse solution generated in the late iteration stage may slow down the algorithm speed due to the excessive position change. Quasi opposition-based learning [27] (QOBL) is a more effective oppositional learning strategy evolved from OBL. It requires that the opposite solution be close to the center M of the search space, not the opposite value; the value of *M* is shown in Equation (17). Figure 1 shows the position schematic of the generated quasi-opposition and the ordinary opposition solutions. It can be seen from the figure that the position of the quasi-opposition solution generated by QOBL is between the opposition solution and the center of the search space.

The expression $X_{i}^{QOBL}$ for the position of the quasi-opposite solution generated by QOBL is shown in Equation (18).

(17) $$M=\left(lb+ub\right)/2$$

(18) $$X_{i}^{QOBL}=\left\{\begin{array}{c} M+\left(X_{i}^{OBL}-M\right)\cdot r,X_{i}<M\\ X_{i}^{OBL}+\left(M-X_{i}^{OBL}\right)\cdot r,else \end{array}\right.$$

where *r* is the uniformly distributed random number belonging to [0, 1].

### 3.3. Elite Steering Factor

Formula (14) describes that crayfish can directly ingest food when the size of the food is appropriate, and there is a certain blindness in this process of crayfish. Therefore, this paper introduces the elite factor [28] to guide the position update, as shown in Equation (19). With its replacement Formula (14), the algorithm can be guided to converge faster and improve the optimization accuracy to a certain extent. The boundary value is taken if the crayfish position updated by Equation (19) is outside the search boundary.

(19) $$X_{i,j}^{t+1}=P\cdot \left(X_{i,j}^{t}-X_{food}\right)+P\cdot r\cdot X_{L}$$

where $X_{L}$ is the optimal position of the current crayfish population, that is the elite.

### 3.4. Vortex Formation and Fish Aggregation Device Effect

Modeling the effects of fish aggregating devices (FADs) [29] was first proposed in the marine predator algorithm. In modeling the FADs effect, the individual position of the population may produce a long jump, which can help the algorithm jump out of the local optimal solution. In this paper, the FADs effect is added to the predation phase of COA. Suppose the crawfish fitness ${fitness}_{i}^{t+1}$ after the update of Equation (19) is not as good as the fitness ${fitness}_{i}^{t}$ of the previous generation, the positions of these crawfish are disturbed according to the FAD effect, and the mathematical expression of this process is shown in (20).

(20) $$X_{i}^{t+1}=\left\{\begin{array}{c} X_{i}^{t}+CF\cdot \left(lb+r\cdot \left(ub-lb\right)\right)\cdot U,\;r\leq FADs\\ X_{i}^{t}+\left(FADs\cdot \left(1-r\right)\cdot \left(X_{r1}^{t}-X_{r2}^{t}\right)\right),\;r>FADs \end{array}\right.$$

where $X_{i}^{t}$ is the current position of the i crayfish, $X_{i}^{t+1}$ represents the position of the next generation, the value of FADs is 0.2, U is a binary vector whose value is between 0 and 1, r is a uniformly distributed random number belonging to [0, 1], and $X_{r1}^{t}$ and $X_{r2}^{t}$ are the positions of two random individuals in the current population.

### 3.5. Pseudo-Code of the ECOA

The pseudo-code of the ECOA proposed in this paper is shown in Algorithm 1.
**Algorithm 1.** The pseudo-code of the ECOAInitialize population size N, number of iterations T, problem dimension D

**for** i = 1:N

  **for** j = 1:D

     Generate the initial population individual position according to Equation (15)

  **end**

**end**
Calculate the fitness value of the population to obtain the values of $X_{G}$ and $X_{L}$

**While** t < T
  Define the ambient temperature Temp through Equation (3)

  **for** i = 1:N

       Crayfish perform QOBL according to Equation (18)

  **end**

  Choosing to retain crayfish populations with better fitness for the next generation

  **if** Temp > 30

    Define the cave location $X_{shade}$ according to Equation (5)

    **if** rand> 0.5

       Crayfish undergo the summer retreat stage according to Equation (6)

    **else**

       Crayfish compete in stages according to Equation (8)

    **end**

  **else**

    Define food intake P and size Q through Equations (4) and (11), respectively

    **if** Q > 2

       Crayfish shred food according to Equation (12)

       Crayfish ingest food according to Equation (13)

    **else**

       Crayfish can directly consume food according to Equation (19)

       **if**
${fitness}_{i}^{t+1}$ < ${fitness}_{i}^{t}$

       The position of crayfish remains unchanged

       **else**

       Update the FADs effect of crayfish based on Equation (20)

       **end**

    **end**

  **end**

Perform boundary processing

Update fitness values, $X_{G}$ and $X_{L}$ values

   t = t + 1

**end**

### 3.6. Analysis of Computational Time Complexity of the ECOA

In the standard crayfish optimization algorithm (COA), N is the number of crayfish populations, D is the number of problem variables, and T is the maximum number of iterations in the algorithm. The time complexity of the standard crayfish optimization algorithm is O(N×D×T). In the ECOA, the complexity of the introduced Halton sequence initialization is O(N×D), which is the same as the original random initialization. The complexity of quasi opposition-based learning is also O(N×D). The elite factor introduced during the predation phase is an improvement on the established steps of the original algorithm and will not increase the complexity of the original algorithm. Due to the fact that only a small number of crayfish undergo the FADs effect, its time complexity is much smaller than O(N×D). In summary, the overall complexity of the ECOA is less than O(N×D×(3+T)). Therefore, the proposed ECOA does not increase much computational complexity and is on the same order of magnitude as the complexity of COA.

## 4. The ECOA Effectiveness Test Experiment

### 4.1. Experimental Scheme

In this section, we conduct simulation experiments on the IEEE CEC2019 [30] test function set to verify the proposed enhanced crayfish optimization algorithm’s (ECOA) optimization performance. The name, dimension D, range and optimal value information of the IEEE CEC2019 test function set are shown in Table 1, which contains 10 minimally optimized single-objective test functions, which is highly challenging.

This paper compares the ECOA with a nine-population intelligent optimization algorithm. They include advanced standard optimization algorithms: the particle swarm optimization algorithm (PSO), the Aquila Optimizer [31] (AO), the Beluga Optimization algorithm [32] (BWO), the golden jackal optimization algorithm [33] (GJO), and the crayfish optimization algorithm (COA). Recently, advanced optimization algorithms have been proposed: the sine-cosine chaotic Harris Eagle Optimization Algorithm [34] (CSCAHHO), the Adaptive slime fungus algorithm [35] (AOSMA), the mixed arithmetic-trigonometric optimization algorithm [36] (ATOA), and the Adaptive Gray Wolf Optimizer [37] (AGWO). These algorithms include the most widely used algorithms, recently proposed algorithms, and four highly advanced improved algorithms. They have demonstrated strong optimization performance in previous research, and compared with these algorithms, they better reflect the excellent optimization ability of the proposed ECOA. The population of all algorithms is set to 30, the maximum number of iterations is set to 2000, and the critical parameters of each algorithm are set using the original algorithm parameters, as shown in Table 2.

All experiments were conducted on a computer with a Windows 10 operating system, a Intel(R) Core (TM) i7-7700HQ 2.80 GHz CPU and 16 GB memory. The simulation experiment platform used is Matlab R2022a. This experiment is divided into five parts: numerical experiment analysis, iterative curve analysis, box plot analysis, the Wilcoxon rank sum tests and ablation experiments. The above experimental system verifies the effectiveness and superiority of the ECOA.

### 4.2. Numerical Experiment and Analysis

In this section, the results of each algorithm running 30 times on CEC2019 are counted, and the statistical indicators include the best value (Best), the mean value (Mean), and the standard deviation (Std). The numerical experimental results of the ECOA and other comparisons are shown in Table 3. In addition to the PSO and the GJO, the ECOA and other algorithms have an optimal value of 1 on F1. The average and optimal values obtained by the ECOA are the minimum, and the minimum standard deviation is obtained on most test functions on F2–F9. By comparing the best value with the average value, it can be seen that the proposed ECOA algorithm has higher optimization accuracy than other algorithms. From the statistical results of the standard deviation, it can be seen that the ECOA proposed in most test functions has higher robustness.

### 4.3. Iterative Curve Analysis

**Figure 2:**
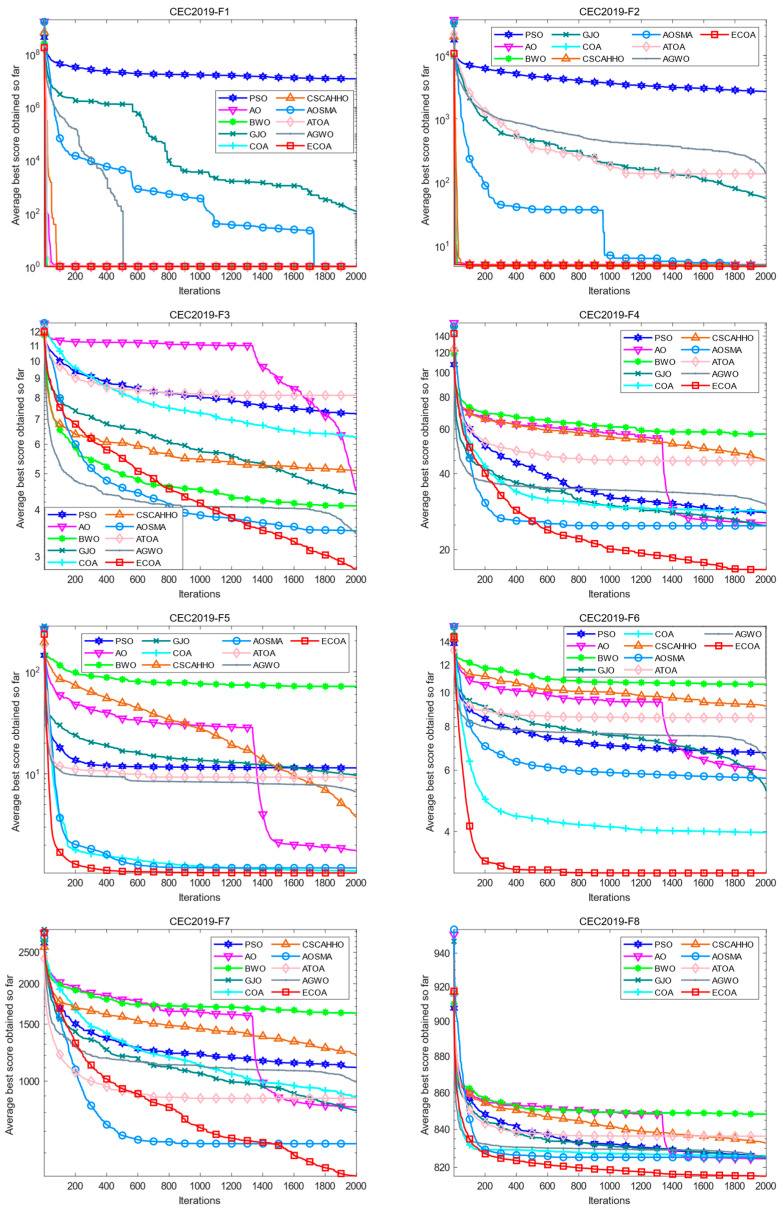

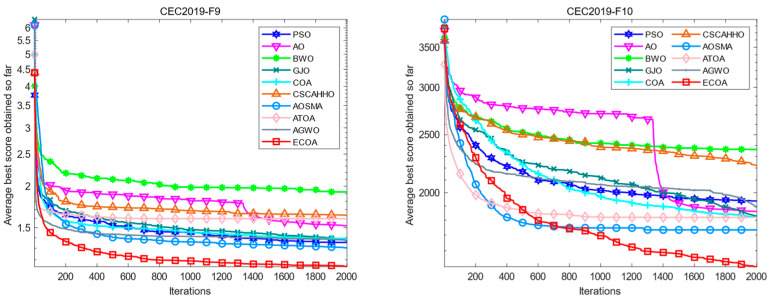
Comparison of iteration curves of each algorithm on the CEC2019 test set.

This paper took the average value of the 30 iterations in which each optimization algorithm was independently run and drew iteration curves for intuitive comparison to verify the proposed ECOA’s optimization performance further. The results are shown in Figure 2. On F1, in addition to the slow convergence speed of PSO and GJO, other algorithms converge quickly to the optimal value, and the ECOA’s convergence speed is the fastest. On F2, except for the slow convergence speed of PSO, GJO, ATOA, and AGWO, other algorithms converge to a quick and close precision. On F3, F4, F7, F8, F9, and F10, although the speed is slightly behind that of some algorithms in the early stage of iteration, other algorithms fall into local optimality with the progress of iteration. The speed is slower, while the ECOA can search and converge faster and has a more vital ability to jump out of local optimality. On both F5 and F6, the ECOA converges to a better value faster than other algorithms. The proposed ECOA has a faster convergence speed and a more vital ability to jump out of local optima compared with the standard optimization algorithm or improved algorithm.

### 4.4. Box Plot Analysis

**Figure 3:**
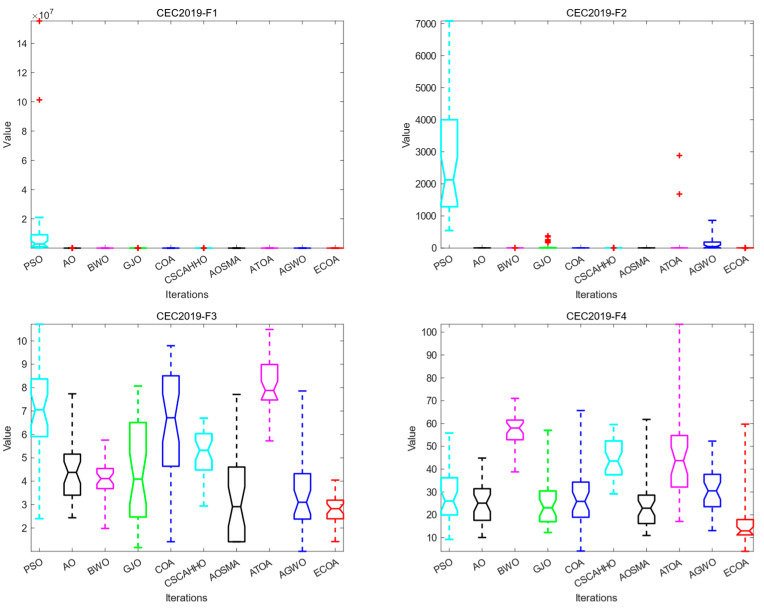

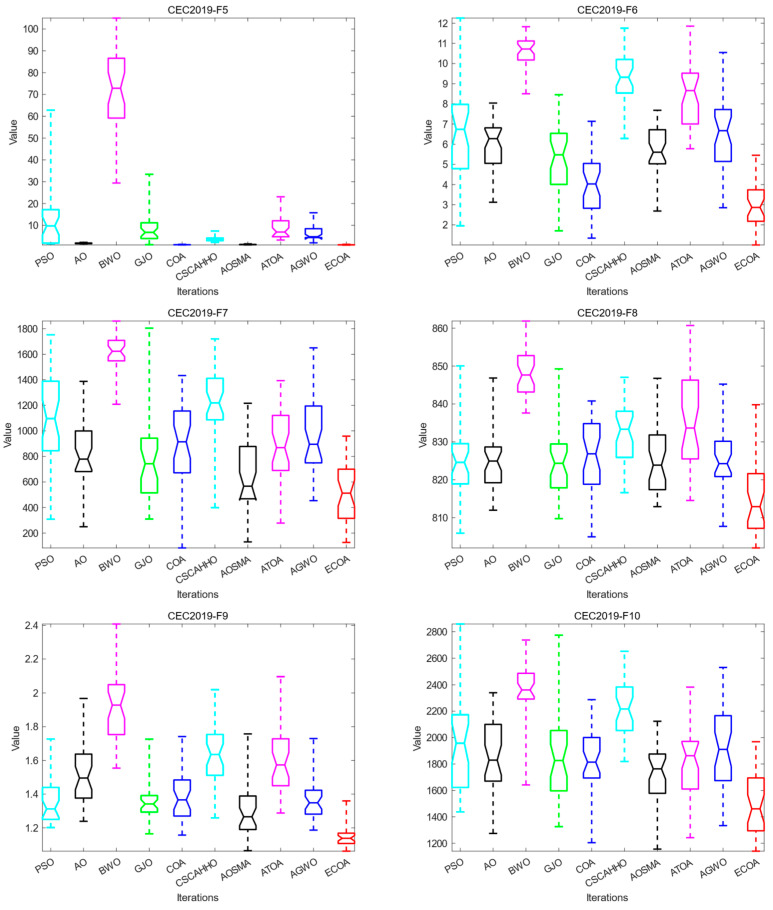
Comparison of box plots of various algorithms on the CEC2019 test set.

We draw boxplots according to the optimization results of the ECOA and other algorithms running 30 times on each test function and carry out the box plot analysis experiment. The boxplot [38] can reflect the distribution and spread range of 30 statistical results for each algorithm and reflect each algorithm’s performance stability when optimizing the IEEE CEC2019 test function. The shorter the box range means that the algorithm’s values are more similar when run several times, and the better the stability and reliability of the algorithm can be obtained. The smaller the value of the median line, the higher the optimization accuracy of the algorithm. The box diagram results are shown in Figure 3. In addition to the results of the ECOA boxplot on F1, F2, and F5, the ECOA’s boxplot range is shorter, and the median line is lower than that of the compared algorithms on other test functions. As a result, the ECOA can achieve better optimization results during multiple runs, and its performance is more stable, which can be used as a more reliable optimization algorithm.

### 4.5. The Wilcoxon Rank Sum Test

The Wilcoxon rank sum test [39] is a nonparametric statistical method that can statistically test the difference in the optimization performance of optimization algorithms. This paper compares the ECOA running 30 times in IEEE CEC2019 with the results of other algorithms by the Wilcoxon rank sum test. The *p* value of the significance level was set at 0.05. Suppose the rank sum test result of the ECOA and the compared algorithm is less than 0.05. In that case, it indicates that the optimization performance of the ECOA is completely better than that of the compared algorithm, and there is a significant difference. Table 4 shows the results of the Wilcoxon rank sum test between the ECOA and each algorithm. It can be seen that the *p*-value of the ECOA and the compared algorithm is less than 0.05 in most of the number functions, which has a significant difference. “N/A” indicates no significant difference between the two algorithms. In the last row of the table, the “worse”, “same”, and “better” of the optimization performance of the ECOA compared with the algorithm are visually counted, which are represented by “–”, “=“, and “+”, respectively. The performance of the ECOA in comparison to the compared algorithms is less than 0.05 in the vast majority of *p*-values, with slight differences only in individual functions (highlighted in bold). The rank sum test verifies that the ECOA has significantly improved performance compared with other algorithms.

### 4.6. Analysis of Ablation Experiments

In this section, in order to further verify whether all four improvement strategies adopted for COA have had a positive improvement effect, ablation experiments are conducted to analyze. In the experiment, we will conduct ablation comparison experiments using only HCOA initialized with the Halton sequence, QCOA with only quasi opposition-based learning strategy, ELCOA with only elite factor, FCOA with only FADs effect, original COA, and the ECOA proposed by the mixed four strategies. We selected eight different types of functions from the widely used and IEEE CEC2017 test function set for ablation experiments to further verify the rationality of the improved strategy and the superiority of the proposed ECOA. The information of eight test functions is shown in Table 5. To maintain fairness, the population size of all algorithms is set to 30 and the maximum number of iterations is set to 2000.

**Figure 4:**
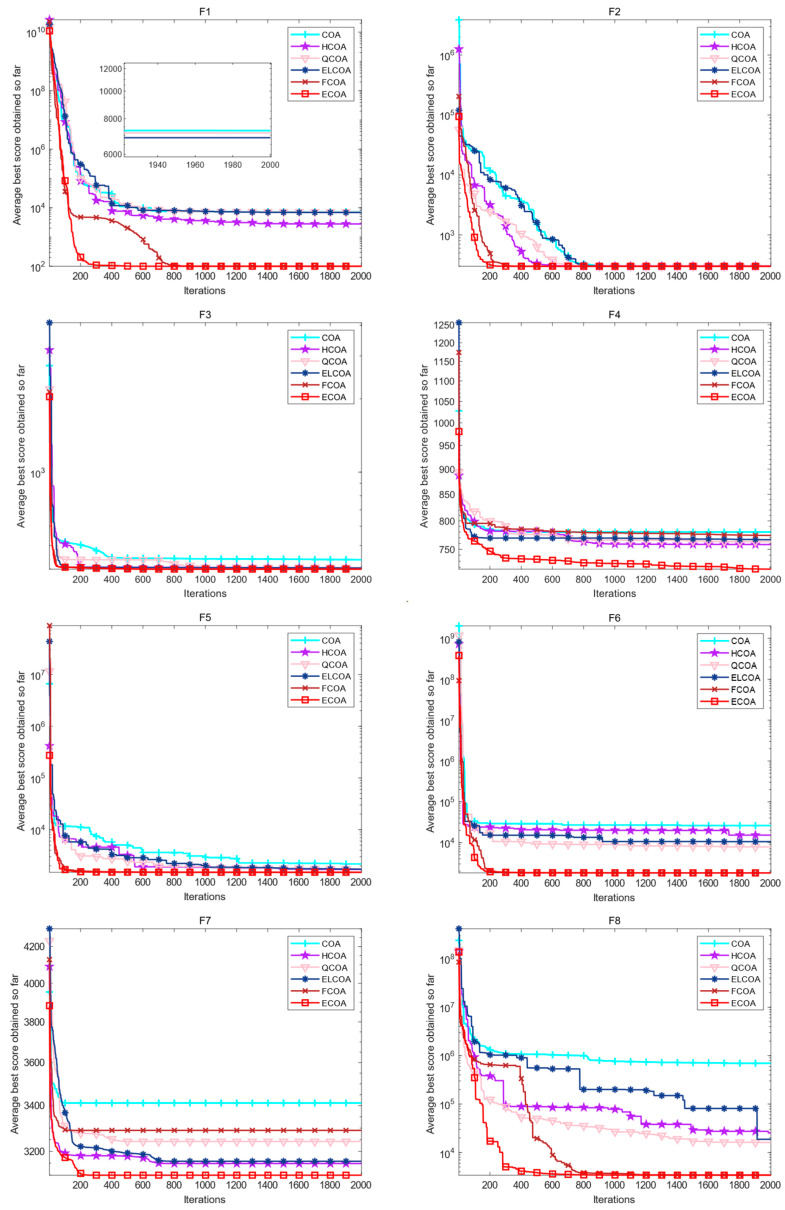
Iteration curves of various algorithms in ablation experiments.

The iteration curve results of all algorithm ablation experiments are shown in Figure 4. The results show that on eight different types of IEEE CEC2017 test functions, each single strategy improved COA has faster convergence speed and optimization accuracy compared to the original algorithm, and the optimization performance of the proposed ECOA is significantly better than other algorithms. Through the above experiments, the feasibility of the adopted strategy was further verified, and mixing these four strategies can comprehensively improve the optimization performance of the original algorithm.

## 5. Practical Engineering Optimization Experiment

In this section, to verify the optimization performance of the proposed ECOA in solving practical engineering problems, we will use the ECOA to solve two engineering design optimization problems [40], namely the three-bar truss design problem and the pressure vessel design problem. Compared with other algorithms, 12 standard and improved algorithms were tested, including the marine predator algorithm (MPA), sine cosine algorithm [41] (SCA), PSO, AO, BWO, GJO, COA, CSCAHHO, AOSMA, ATOA, and AGWO. All algorithms run independently 30 times to statistically analyze the experimental results, with a population size of 30 and a maximum iteration count of 2000.

### 5.1. Three-Bar Truss Design Problem

**Figure 5:**
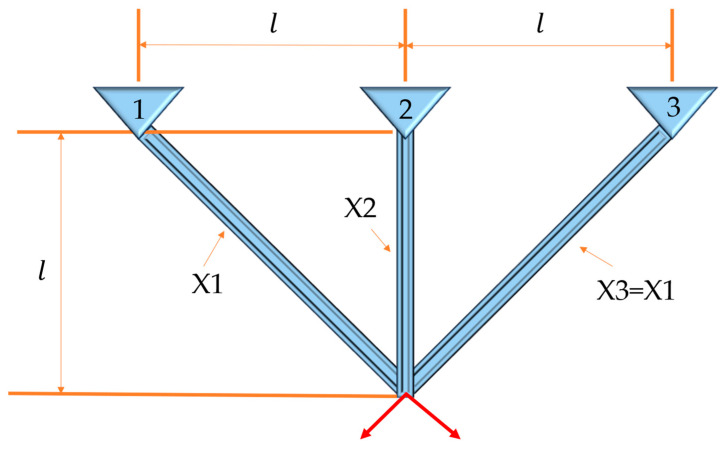
Three-bar truss structure diagram.

**Figure 6:**
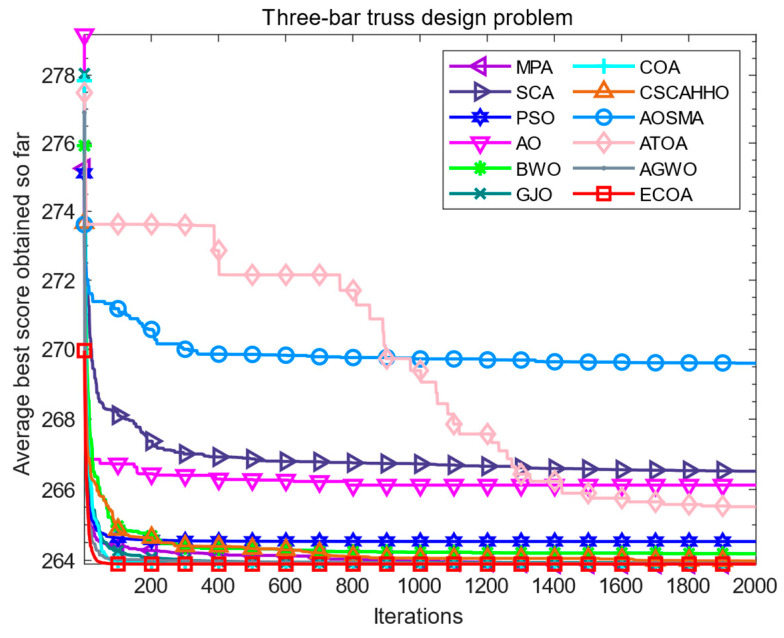
Iterative curves of various algorithms for three-bar truss design problems.

The three-bar truss design problem refers to the minimization of the volume of the truss by optimizing and adjusting the cross-sectional areas (X1 and X2) under stress constraints on each truss. The structure of the three-bar truss is shown in Figure 5, l represents spacing, X1, X2, and X3 represent cross-sectional areas. Its objective function is nonlinear and includes two decision parameters and three inequality constraints. The fitness iteration curve results of all algorithms are shown in Figure 6, which shows that compared to other algorithms, the ECOA has a faster convergence speed and higher convergence accuracy. The numerical results of each algorithm are summarized in Table 6. The ECOA results in higher accuracy and more stable performance.

### 5.2. Pressure Vessel Design

**Figure 7:**
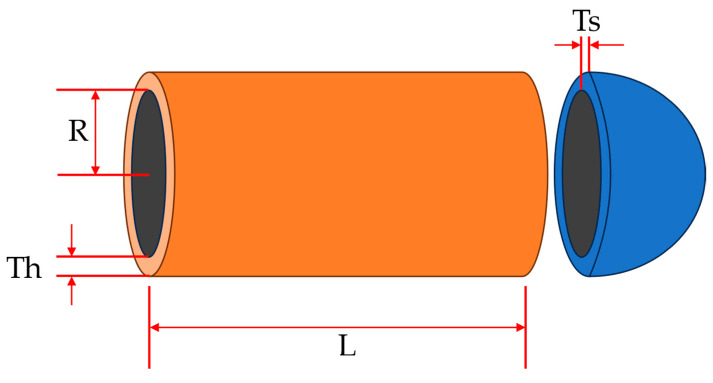
Schematic diagram of pressure vessel structure.

**Figure 8:**
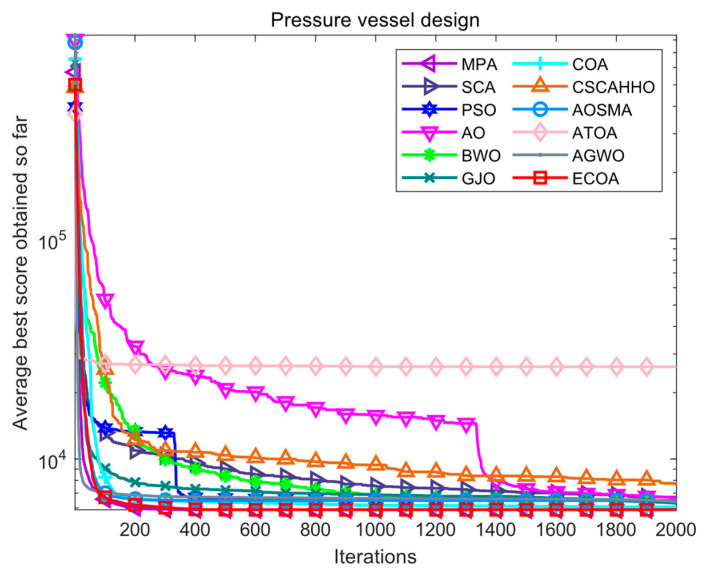
Iterative curves of various algorithms for pressure vessel design.

The pressure vessel design problem is to obtain the design structure of a pressure vessel with minimal cost through optimization while satisfying constraints. There are four decision variables that need to be optimized for this problem, namely: hemispherical head thickness (Th), container wall thickness (Ts), cylindrical section length (L), and inner diameter (R). The structural diagram of the pressure vessel is shown in Figure 7. The fitness iteration curves of all algorithms for pressure vessel design problems are shown in Figure 8. The optimization results of various algorithms for pressure vessel design problems are shown in Table 7. Based on the results in Figure 8 and Table 7, it can be seen that the results obtained by the ECOA and MPA are superior to those of other algorithms, and both have achieved optimal results. Moreover, from the convergence curve, it can be seen that the ECOA quickly converged to a better value, indicating its feasibility and superiority in applying to this problem.

## 6. Conclusions and Future Work

The crayfish optimization algorithm (COA) is a new swarm intelligence optimization algorithm that is performing well in global and engineering optimization. However, the COA also has some defects. In this paper, the standard COA mixing strategy is improved: (1) The Halton sequence is used to initialize the population so that the initial population is more evenly distributed in the search space, and the convergence speed of the initial COA iteration is increased. (2) QOBL is introduced to generate the quasi-oppositional solution of the population. The better solution is selected from the original solution, and the quasi-oppositional solution enters the next generation to improve the quality of candidate solutions. (3) Introducing elite guiding factors in the predation stage to avoid the blindness of this process. (4) The fish device aggregation effect (FADs) in the marine predator algorithm is introduced into COA to enhance its ability to jump out of local optimal. Based on the above, an enhanced crayfish optimization algorithm (ECOA) with better performance is proposed. This paper compares it with other optimization algorithms and improved algorithms on the CEC2019 test function and two real-world engineering optimization problems set to verify the effectiveness of the ECOA. The experimental results show that the ECOA can better balance the exploration and development of the algorithm and has a more robust global optimization ability. In future work, we will apply the ECOA to other practical problems, such as UAV track optimization, flexible shop scheduling, and microgrid optimization scheduling, to verify its ability to solve various complex practical problems.

## Figures and Tables

**Table 1:** Information of IEEE CEC2019 test function set.

Function	Name	D	Search Range	Optimum
F1	Storn’s Chebyshev Polynomial Fitting Problem	9	[−8192, 8192]	1
F2	Inverse Hilbert Matrix Problem	16	[−16,384, 16,384]	1
F3	Lennard–Jones Minimum Energy Cluster	18	[−4, 4]	1
F4	Rastrigin’s Function	10	[−100, 100]	1
F5	Griewangk’s Function	10	[−100, 100]	1
F6	Weierstrass Function	10	[−100, 100]	1
F7	Modified Schwefel’s Function	10	[−100, 100]	1
F8	Expanded Schaffer’s F6 Function	10	[−100, 100]	1
F9	Happy Cat Function	10	[−100, 100]	1
F10	Ackley Function	10	[−100, 100]	1

**Table 2:** Important parameter settings of each algorithm.

Algorithms	Parameters
PSO	ω = 0.9, C1 = C2 = 2
AO	α =0.1, δ = 0.1
BWO	Probability of whale fall decreased at interval $W_{f}\in [0.1,\;0.05]$
GJO	$E_{0}\in [-1,\;1]$
COA	r∈[0, 1]$,\;C_{2}=2-\left(t/T\right)$$,\;C_{3}=3$,
CSCAHHO	a=2 , r, r1∈[0, 1] , r2∈[0, 2π] ,r3∈[0, 2] , E1∈[2, 0]
AOSMA	δ = 0.03
ATOA	min=0.2, max=1, α=5 , μ=0.499 , ε=1
AGWO	γ damping when F is not decreasing significantly
ECOA	FADs = 0.2

**Table 3:** Numerical experimental results of each algorithm on the CEC2019 test set.

CEC2019	Value	PSO	AO	BWO	GJO	COA	CSCAHHO	AOSMA	ATOA	AGWO	ECOA
F1	Best	1.361 × 10^+5^	1	1	1	1	1	1	1	1	1
	Mean	1.202 × 10^+7^	1	1	120.2597	1	1	1	1	1	1
	Std	3.027 × 10^+7^	1.135 × 10^−9^	0	642.788	0	9.879 × 10^−11^	0	0	0	0
F2	Best	545.9192	5	4.8556	4.2197	4.0557	4.2739	4.2316	4.4755	4.2174	3.2141
	Mean	2689.1055	5	4.9935	55.3157	4.8087	4.9449	4.7893	135.0859	133.871	4.6568
	Std	1741.3001	0	0.027086	106.4349	0.33374	0.17328	0.32882	556.0031	194.2698	0.64381
F3	Best	2.3979	2.4361	1.9805	1.1634	1.4104	2.9411	1.4091	5.7234	1.0004	1.4173
	Mean	7.2501	4.4884	4.103	4.4136	6.2606	5.0994	3.517	8.1086	3.462	2.7698
	Std	1.9616	1.2868	0.766	2.2871	2.6603	1.0752	2.2299	1.2277	1.532	0.62451
F4	Best	9.2209	10.0576	38.8009	12.248	4.1414	29.1901	10.9496	17.0898	13.0603	3.9849
	Mean	27.9446	25.5342	57.3153	24.843	28.4397	44.8217	24.8393	44.8809	30.3264	16.6629
	Std	11.0734	8.7901	6.9365	10.7428	15.7463	8.8591	11.3231	16.7632	9.5238	11.1345
F5	Best	1.5511	1.4833	29.405	1.1339	1.0497	2.1918	1.0246	3.2675	1.9698	1.0074
	Mean	11.428	1.7597	72.0211	9.5542	1.115	3.7237	1.194	9.2733	6.6392	1.0641
	Std	13.5064	0.18663	19.4782	9.0179	0.061305	1.1233	0.11646	5.8361	3.8098	0.050987
F6	Best	1.9552	3.1214	8.5015	1.7038	1.3443	6.2846	2.6872	5.7768	2.8508	1.0041
	Mean	6.7367	5.9982	10.5902	5.2258	3.9784	9.1707	5.6918	8.4921	6.4251	3.0444
	Std	2.4593	1.2484	0.77804	1.7237	1.5453	1.3384	1.3033	1.6149	1.7536	1.268
F7	Best	308.3668	249.3434	1208.3006	309.9122	83.4932	399.241	130.7135	278.2579	453.8124	126.6386
	Mean	1102.4747	832.3015	1623.6728	810.9943	894.644	1206.1076	640.6508	884.6521	994.0343	508.5085
	Std	355.712	265.7038	130.0862	376.0119	339.8851	290.3894	277.6008	269.8459	298.1859	230.6677
F8	Best	805.925	811.9902	837.6275	809.7835	804.9748	816.6299	812.9345	814.5553	807.7328	802.0457
	Mean	825.7583	824.5015	848.2828	824.7693	825.9996	832.6092	825.2471	836.4446	825.521	815.3867
	Std	10.1005	7.4753	6.0262	9.2526	8.9454	8.4370	8.9263	13.1182	7.8571	9.9757
F9	Best	1.1616	1.1532	1.5475	1.2025	1.1683	1.3514	1.0538	1.1977	1.2129	1.0359
	Mean	1.3579	1.4924	1.9341	1.3228	1.3786	1.6591	1.2842	1.6030	1.3956	1.1378
	Std	0.0999	0.1697	0.1336	0.0928	0.1442	0.1545	0.1223	0.2205	0.1129	0.0522
F10	Best	1436.99	1274.08	1641.75	1325.81	1204.19	1818.69	1155.34	1241.95	1333.24	1139.99
	Mean	1937.94	1861.89	2362.49	1828.64	1819.19	2217.23	1733.81	1819.03	1892.962	1505.39
	Std	349.35	275.94	194.21	344.89	267.68	216.92	214.91	268.69	309.42	229.69

**Table 4:** Test *p*-values of each algorithm on the IEEE CEC2019 test set.

CEC2019	PSO	AO	BWO	GJO	COA	CSCAHHO	AOSMA	ATOA	AGWO
F1	1.534 × 10^−14^	3.706 × 10^−4^	N/A	1.534 × 10^−14^	N/A	2.527 × 10^−10^	N/A	N/A	N/A
F2	1.322 × 10^−13^	3.017 × 10^−3^	3.031 × 10^−3^	2.943 × 10^−2^	7.786 × 10^−1^	3.253 × 10^−1^	9.044 × 10^−1^	8.363 × 10^−2^	1.162 × 10^−7^
F3	5.851 × 10^−12^	2.255 × 10^−8^	2.789 × 10^−9^	7.939 × 10^−3^	2.678 × 10^−7^	1.438 × 10^−11^	8.325 × 10^−1^	6.545 × 10^−13^	5.119 × 10^−2^
F4	6.466 × 10^−6^	7.221 × 10^−6^	3.865 × 10^−12^	6.834 × 10^−5^	6.189 × 10^−5^	9.661 × 10^−11^	7.922 × 10^−5^	2.254 × 10^−10^	1.955 × 10^−7^
F5	6.545 × 10^−13^	6.545 × 10^−13^	6.545 × 10^−13^	7.132 × 10^−13^	2.477 × 10^−5^	6.545 × 10^−13^	2.248 × 10^−9^	6.545 × 10^−13^	6.545 × 10^−13^
F6	4.928 × 10^−9^	2.625 × 10^−10^	6.545 × 10^−13^	1.156 × 10^−6^	1.044 × 10^−2^	6.545 × 10^−13^	1.565 × 10^−9^	6.545 × 10^−13^	2.625 × 10^−10^
F7	7.510 × 10^−10^	6.118 × 10^−6^	6.545 × 10^−13^	5.536 × 10^−4^	2.204 × 10^−6^	2.527 × 10^−11^	6.689 × 10^−2^	4.399 × 10^−7^	1.307 × 10^−8^
F8	1.413 × 10^−4^	9.620 × 10^−5^	7.761 × 10^−13^	2.363 × 10^−4^	2.608 × 10^−5^	1.399 × 10^−8^	5.327 × 10^−5^	8.626 × 10^−9^	3.935 × 10^−5^
F9	5.851 × 10^−12^	2.342 × 10^−12^	6.545 × 10^−13^	4.566 × 10^−12^	5.388 × 10^−12^	6.545 × 10^−13^	2.761 × 10^−8^	1.191 × 10^−12^	1.411 × 10^−12^
F10	5.620 × 10^−7^	1.852 × 10^−6^	1.819 × 10^−12^	4.357 × 10^−5^	4.628 × 10^−6^	2.342 × 10^−12^	1.348 × 10^−4^	9.498 × 10^−6^	1.090 × 10^−6^
−/=/+	0/0/10	0/0/10	0/1/9	0/0/10	0/2/8	0/1/9	0/4/6	0/2/8	0/2/8

**Table 5:** Information on the eight test functions of IEEE CEC2017.

Function Type	Function Number	Name	Dimension	Theoretical Optimal Value
Unimodal function	CEC2017-F1	Shifted and rotated bent cigar function	10	100
	CEC2017-F2	Shifted and rotated zakharov function	10	300
Multimodal function	CEC2017-F3	Shifted and rotated Rosenbrock’s function	10	400
	CEC2017-F4	Shifted and rotated lunacek Bi_Rastrigin	10	700
Hybrid function	CEC2017-F5	Hybrid function 5 (N = 4)	10	1500
	CEC2017-F6	Hybrid function 6 (N = 5)	10	1800
Composition function	CEC2017-F7	Composition function 8 (N = 6)	10	2800
	CEC2017-F8	Composition function 10 (N = 3)	10	3000

**Table 6:** Experimental results of various algorithms on the three-bar truss design problems.

Algorithms	Decision Variables		Best	Mean	Std
	X1	X2			
MPA	0.788543661	0.408621746	263.8958	263.8960	2.2142 × 10^−4^
SCA	0.817087569	0.354123484	263.9162	266.5196	6.5123
PSO	0.795645656	0.394874029	263.8959	264.5300	3.4587
AO	0.759197479	0.513909988	264.0598	266.1245	2.0766
BWO	0.789321915	0.409324881	263.8969	264.1864	0.2262
GJO	0.789507917	0.405951715	263.8961	263.9017	0.0048061
COA	0.788604706	0.408449023	263.8959	263.8960	1.3825 × 10^−4^
CSCAHHO	0.790860857	0.402928576	263.8959	263.9821	0.11688
AOSMA	0.735818117	0.614832778	264.1893	269.6041	2.1968
ATOA	0.822192305	0.329610280	263.9000	265.5121	2.2217
AGWO	0.788828845	0.407890221	263.8972	263.9035	0.0067363
ECOA	0.788675135	0.408248289	263.8958	263.8958	1.7345 × 10^−13^

**Table 7:** Experimental results of various algorithms in pressure vessel design.

Algorithms	Decision Variables				Best	Mean	Std
	Th	Ts	L	R			
MPA	0.384649163	0.778168641	200	40.31961872	5885.3328	5885.3328	5.3255 × 10^−13^
SCA	0.499291880	0.925842231	146.4048851	46.38243053	5984.1873	6717.5887	563.3545
PSO	0.485368191	0.971227746	119.8348813	50.22748055	5887.8888	6471.8242	667.9827
AO	0.507963654	1.000199547	101.8941606	51.19045001	6099.8767	6649.8309	433.1601
BWO	0.475536871	0.888263455	155.1896217	44.84373326	5936.4947	6469.8463	340.4535
GJO	0.461282896	0.928624719	132.504745	48.0893052	5890.1495	6298.0075	527.7598
COA	0.42204187	0.846150966	161.1823893	43.80603079	5886.7775	6050.5243	202.9679
CSCAHHO	0.609416381	1.236751933	46.25754118	59.24840347	6085.6621	7694.8483	900.2840
AOSMA	0.492061006	0.995469322	103.7860047	51.57872133	5890.4997	6475.3967	587.2016
ATOA	0.865903331	1.885676384	51.10292644	84.14450431	6843.7044	26,244.2365	26,733.8240
AGWO	0.473362091	0.950451055	124.8342301	49.17678664	5895.6772	6376.1675	567.2517
ECOA	0.384649163	0.778168641	200	40.31961872	5885.3328	5885.3328	1.6889 × 10^−13^

## Data Availability

The data that support the findings of this study are available from the corresponding author upon reasonable request.
